# CREB promotes starvation-induced sleep loss by regulating octopamine production in *Drosophila*

**DOI:** 10.1242/bio.062570

**Published:** 2026-08-21

**Authors:** Shiye Zhu, Zhian Rao, Youjie Yin, Shuxin Chen, Hansong Deng

**Affiliations:** Department of Gastroenterology, Yangzhi Rehabilitation Hospital, Sunshine Rehabilitation Center, Frontier Science Center for Stem Cell Research, School of Life Sciences and Technology, Tongji University, Shanghai 20092, China

**Keywords:** Starvation, CREB, Octopamine, Huntington's disease, *Drosophila*

## Abstract

Starvation induces robust food-seeking behavior in animals as a fundamental survival response. In *Drosophila*, biogenic amine octopamine (OA) plays a critical role in starvation-induced locomotor hyperactivity and awakeness, while the underlying signaling cascade remains not fully understood. Here, we demonstrated that the conserved transcription factor cAMP-Responsible Element Binding Protein (CREB), activated during starvation through the SIK2-CRTC pathway, contributes to starvation-induced sleep reduction. Mechanistically, we found that tyramine β-hydroxylase, the rate-limiting enzyme for OA synthesis, is upregulated by starvation in a CREB-dependent manner. Selective silencing of CREB in OA-producing neurons ameliorated OA-induced sleep suppression, and *CREB* loss-of-function mutations can partially rescue sleep fragmentation in a *Drosophila* model of Huntington's disease. Collectively, these findings reveal a novel regulatory role for CREB in sleep homeostasis through the transcriptional control of OA biosynthesis.

## Teaser

**Summary:** Hunger defeats sleep: CREB hijacks octopamine synthesis via tyramine β-hydroxylase, forcibly awakening flies to seek food.

## INTRODUCTION

Sleep is a fundamental physiological state that plays a critical role in maintaining physical health and regulating essential biological processes, including metabolic and energy regulation, immune modulation, and cognitive processes (Brown et al., 2012; Diekelmann and Born, 2010).

The *Drosophila* genome encodes counterparts to the majority of mammalian neurotransmitter systems that regulate sleep and circadian rhythms (Crocker et al., 2010; Li et al., 2021; Bargiello et al., 1984), making it an ideal model for studying the molecular mechanisms of sleep regulation.

Foraging behavior is fundamental to the fitness and survival of animals. In *Drosophila*, food deprivation induces locomotor hyperactivity associated with food searching through a signaling pathway involving the peptide adipokinetic hormone (AKH) and the bioactive monoamine octopamine (OA). AKH is produced and released by endocrine cells in the corpora cardiaca (CCA; an analog of mammalian pancreatic alpha cells) and is required to maintain hemolymph sugar and lipid levels (van der Horst et al., 2001). Upon starvation, carbohydrate levels in the hemolymph decrease, promoting the release of AKH from CCA (Held et al., 2025; Ahmad et al., 2025).

OA regulates various behaviors, including sleep (Crocker and Sehgal, 2008), learning (Burke et al., 2012), and aggression (Hoyer et al., 2008). Recent studies have shown that the AKH receptor (AkhR) is expressed in a small group of brain octopaminergic neurons. Silencing these AkhR-expressing neurons or blocking OA signaling within them suppresses starvation-induced hyperactivity (Yu et al., 2016; Yang et al., 2015).

cAMP-Responsible Element Binding Protein (CREB) is a conserved transcription factor that regulates various metabolic and developmental processes (Sakamoto et al., 2011; Wang et al., 2008; Yin et al., 1994; Ma et al., 2024; Meng et al., 2025). It receives upstream signaling inputs, such as cAMP and calcium, and its activity is regulated by phosphorylation (Shaywitz and Greenberg, 1999). Phosphorylated CREB translocates to the nucleus, where it is coactivated by CREB-responsible transcriptional coactivator (CRTC) and CREB-binding protein (CBP)/p300 (Screaton et al., 2004; Chrivia et al., 1993). Activated CREB is then recruited to target genes containing CREB responsible elements (CRE) (Altarejos and Montminy, 2011; Chrivia et al., 1993). In mammals, cAMP signaling and CREB activity are associated with waking status (Cirelli et al., 1996). cAMP accumulation is reduced during sleep deprivation and replenished during recovery in a sleep-related area of the hypothalamus (Zamboni et al., 1999). However, the causal relationship between CREB activity and sleep remains unclear, and the underlying molecular mechanisms are poorly understood.

Sleep deprivation leads to acute impairments in cognition, motor function, emotion and even lethality (Yoo et al., 2007; Zhou et al., 2008). Chronic sleep disturbances are further associated with behavioral dysregulation and, based on animal studies, may contribute directly to neurodegeneration (Musiek and Holtzman, 2016). For instance, sleep disruption is a prominent non-motor symptom in Huntington's disease (HD), a progressive neurodegenerative disorder caused by a CAG repeat expansion in the *Htt* gene (Morton, 2013). In HD fly models, sleep and activity disruptions emerge early in adulthood (Gonzales and Yin, 2010; Faragó et al., 2019). Notably, elevating PKA/CREB activity in healthy flies recapitulates these HD-like sleep patterns, suggesting a shared mechanistic pathway (Hendricks et al., 2001). However, the mechanisms of how CREB regulates sleep are yet to be elucidated.

Here, we demonstrate that starvation activates CREB to suppress sleep by upregulating tyramine β-hydroxylase (TβH) expression. Notably, inhibiting CREB rescued sleep disturbances in a *Drosophila* HD model, identifying CREB hyperactivity as a critical mediator of sleep dysfunction across conditions.

## RESULTS

### CREB is activated in the *Drosophila* brain during starvation-induced sleep loss

**Fig. 1.**
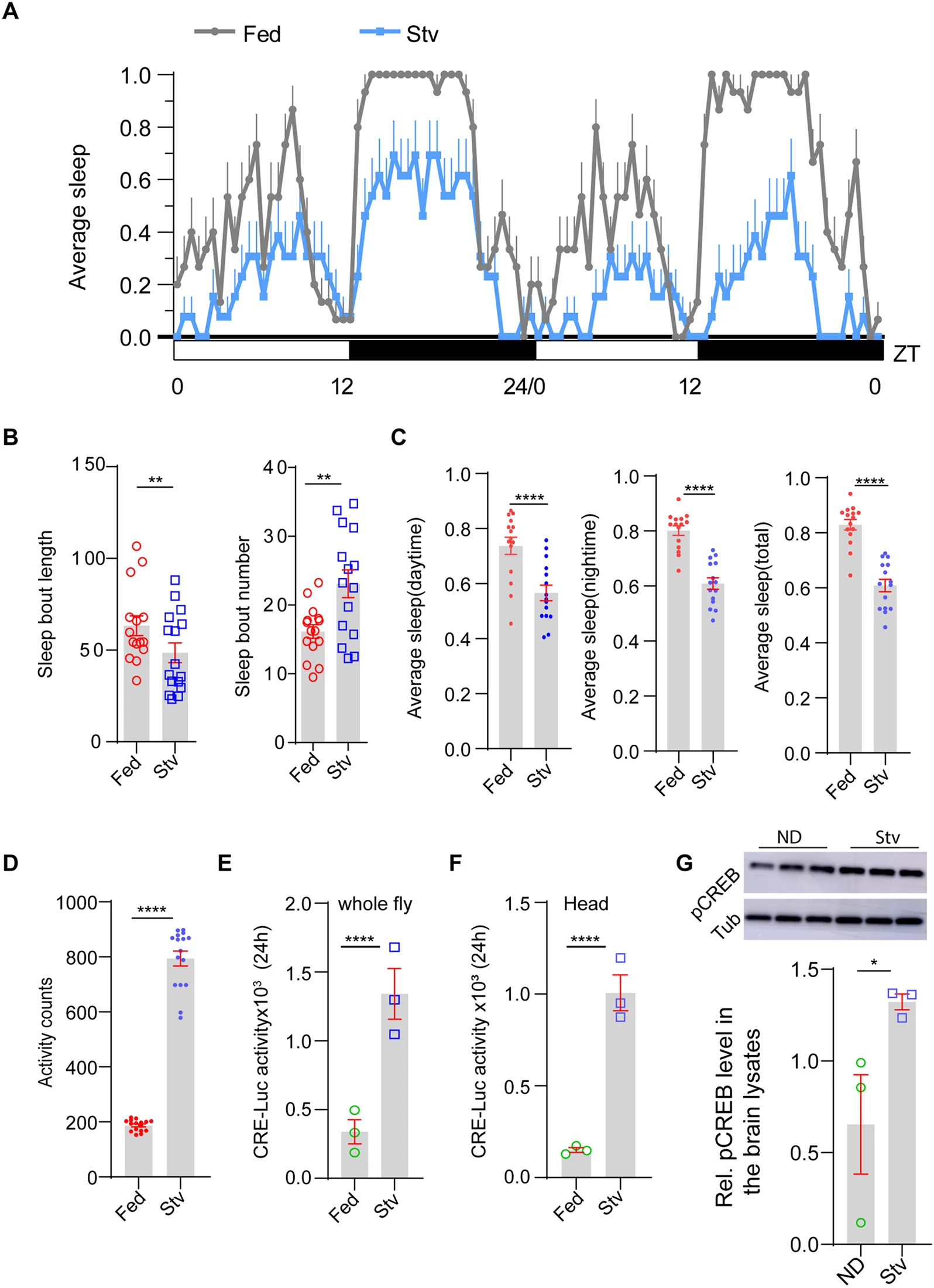
**CREB is activated in the *Drosophila* brain during starvation-induced sleep loss.** (A) Sleep profiles are shown upon starvation (Stv) treatment. The average sleep ratio is shown on the *Y*-axis, and the ZT in h is on the *X*-axis (*n*=12 animals for each group). (B) The average length and number of sleep bouts were quantified in flies. (C) Daytime, nighttime and total average sleep ratios were quantified. For B and C, mean±s.e.m. are shown, *t*-test for statistical analysis. ****P<0.0001 (*n*=12 animals for each group). (D) Locomotor activity (beam crosses/day) was analyzed upon starvation (*n*=12 animals for each group). Biological triplicates were performed; mean±s.e.m. are shown, *t*-test for statistical analysis. ****P<0.0001. (E,F) Luciferase activity was examined in whole bodies or heads of flies carrying CRE-Luc under fed or starved conditions. Relative luciferase activity was normalized to total protein levels. Biological triplicates were analyzed. Mean±s.e.m. are shown, *t*-test for statistical analysis. ****P<0.0001. Genotype for E and F: *5xCRE-Luc*, and for the remaining panels: *w^1118^*. (G) CREB activity was examined in brain lysates via immunoblotting with an anti-p-CREB antibody, and relative band intensities were normalized to α-tubulin as an internal loading control. Quantifications were shown on the right. Mean±s.e.m. are shown, *t*-test for statistical analysis. *P<0.05, **P<0.01, ****P<0.0001.

Starvation induced by 24-h exposure to double-distilled H_2_O significantly altered sleep architecture, reducing both the number and duration of sleep bouts during the day and night (Fig. 1A-C) and increasing locomotor hyperactivity (Fig. 1D). To assess CREB transcriptional activity, we utilized flies expressing a CRE-Luc reporter, in which luciferase expression is driven by five copies of CRE sites (Wang et al., 2025; Yin et al., 2022). As shown in Fig. 1E,F, luciferase activity was significantly elevated in whole-fly extracts as well as in fly heads upon starvation. Meanwhile, western blot with an antibody against pCREB also significantly increased in brain lysates upon starvation (Fig. 1G). These data indicated that CREB transcriptional activity is robustly upregulated in *Drosophila* heads under starvation conditions.

### Starvation-induced sleep deprivation relies on CREB

We next examined the role of CREB in starvation-associated sleep deprivation.

**Fig. 2.**
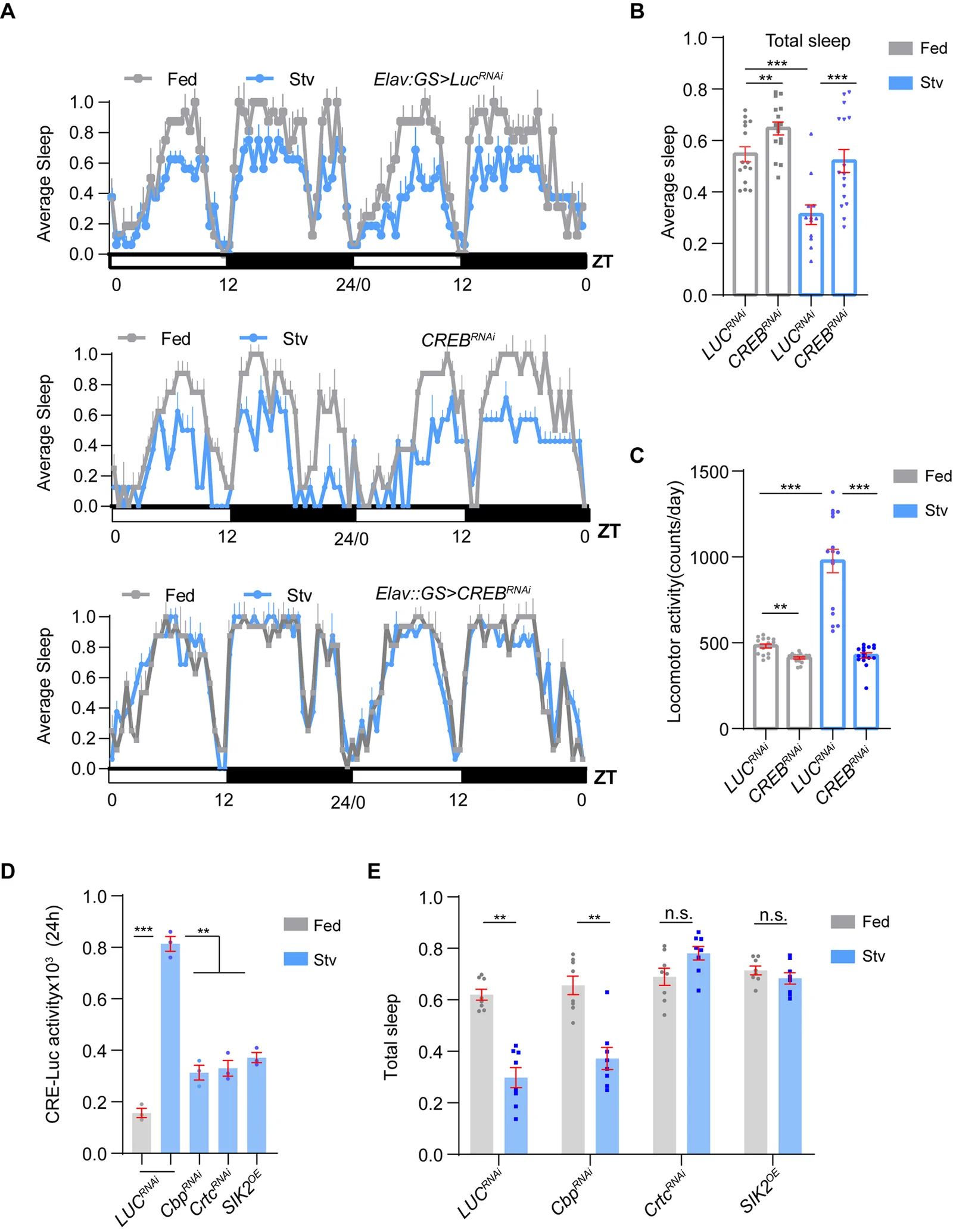
**Starvation-induced sleep deprivation relies on CREB.** (A-C) Sleep profiles with indicated genotypes were shown. Average total sleep rate (B) and locomotor activity beam crosses per day (C) were compared with different genotypes. (D) Relative luciferase activity of CRE-Luc was quantified in fly heads of different genotypes. (E) Total sleep ratio in 24 h was quantified with indicated genotypes. Biological triplicates were performed, one-way ANOVA for B and C and two-way ANOVA analysis for D and E. Mean±s.e.m. are shown. **P<0.01, ***P<0.001, n.s.: no significance (*n*=12 animals for each group). Genotypes for A-C: *Elav-GS-Gal4; UAS-Luc^RNAi^* or *UAS-CREB^RNAi^* or *Elav-GS-Gal4; UAS-CREB^RNAi^*. For D and E: *Elav-GS-Gal4; UAS-Luc^RNAi^* or *Elav-GS-Gal4; UAS-Cbp^RNAi^* or *Elav-GS-Gal4; UAS-Crtc^RNAi^* or *Elav-GS-Gal4; UAS-SIK2^OE^*.

As shown in Fig. 2A-C and Fig. S1A-C, pan-neuronal silencing of *CREB* using the drug-inducible *Elav-GS*-GAL4 driver after 2-day RU486 feeding significantly reduced sleep rate in a 24-h Zeitgeber time (ZT) period while increasing locomotor activity. We then sought to test how CREB was activated during starvation. CRTC (also known as TORC) functions as a coactivator of CREB, and its activity is regulated by phosphorylation-dependent nucleocytoplasmic shuttling. When phosphorylated by salt-inducible kinases (SIKs), CRTC is sequestered in the cytosol through interaction with 14-3-3 proteins and is targeted for degradation. In contrast, the dephosphorylated form translocates into the nucleus and acts as a CREB coactivator (Screaton et al., 2004; Wang et al., 2008). Notably, SIK2 activity is suppressed during starvation (Wang et al., 2008), a condition expected to relieve CRTC inhibition and promote CREB activation. Consistent with this model, neuronal knockdown of *CBP* or *CRTC*, as well as neuronal overexpression of SIK2, significantly reduced starvation-induced CREB activity (Fig. 2D). Interestingly, the resulting starvation-induced sleep deprivation was restored by *CRTC^RNAi^* or *SIK2^OE^*, while *CBP^RNAi^* failed to provide a rescuing effect (Fig. 2E; Fig. S1D). Together, these results indicate that CREB activation via the SIK2/CRTC cascade is required for starvation-induced sleep loss.

### CREB activity in AkhR-positive neurons is critical for starvation-associated sleep loss

**Fig. 3.**
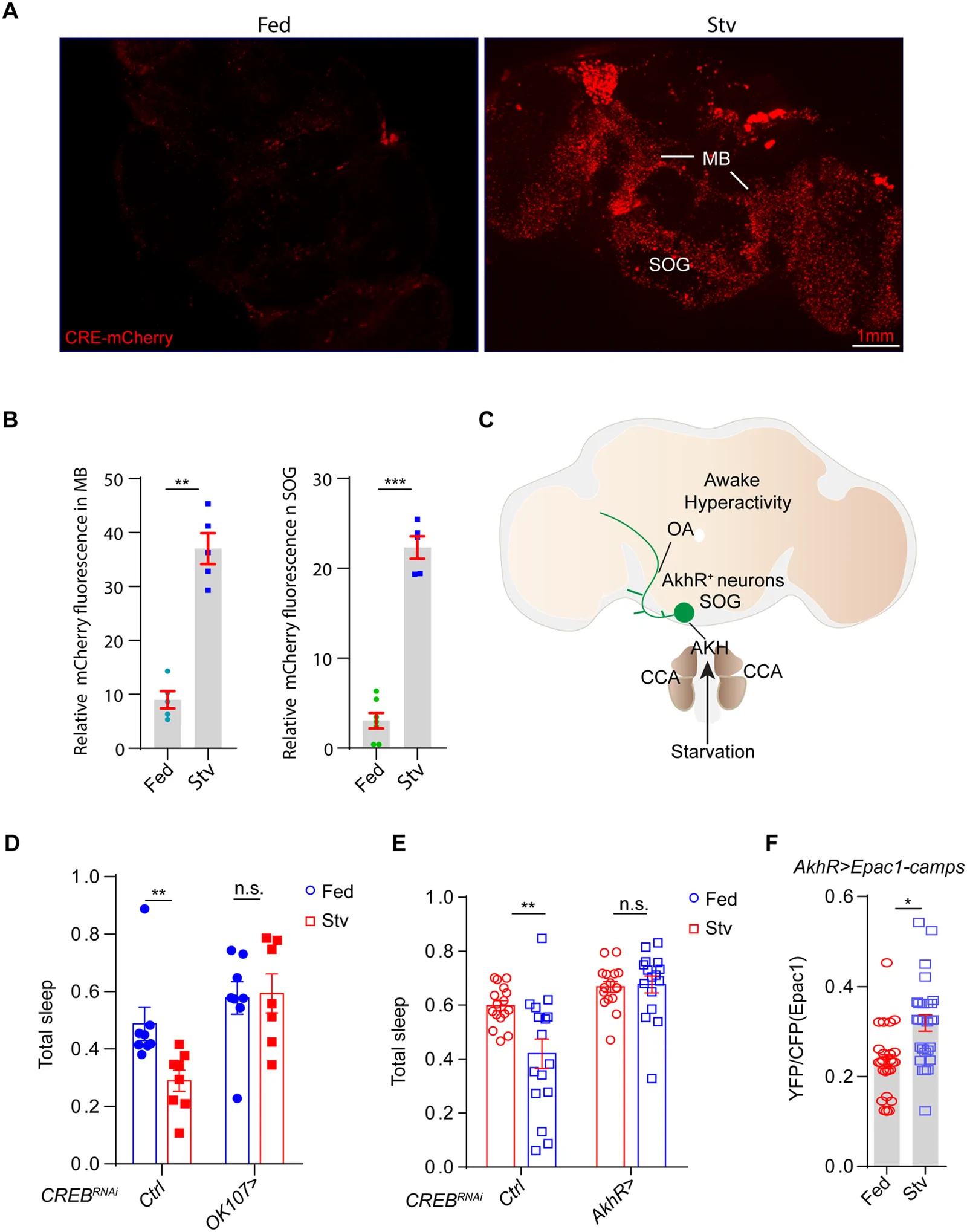
**CREB activity in AKHR-positive neurons is critical for starvation-associated sleep loss.** (A,B) CREB activity in brains (*ex vivo*) was monitored using a 5xCRE-mCherry reporter. A significant induction of the reporter was observed in the SOG and MB of starved (Stv) animals compared to fed controls. Scale bars: 1 mm. Quantification was shown in B. Each dot represents one animal (*n*=5). Mean±s.e.m. are shown, *t*-test for statistical analysis. **P<0.01, ***P<0.001. (C) Simplified neuronal circuits regulate starvation-induced wakefulness and locomotion. Starvation triggers the release of the glucagon-like peptide AKH from the CCA. AKH then acts on its receptor (AkhR) in neurons of the SOG, inducing OA production. Finally, OA promotes both wakefulness and locomotor activity. (D) CREB knockdown in the MB using OK107-Gal4 was sufficient to block the sleep suppression caused by starvation. Mean±s.e.m. are shown, two-way ANOVA for statistical analysis. **P<0.01, n.s.: no significance (*n*=12). Genotypes: *OK107-Gal4; UAS-CREB^RNAi^* or *OK107-Gal4*. (E) Total sleep was measured following CREB inhibition specifically in AkhR-positive neurons. Two-way ANOVA for statistical analysis. **P<0.01, n.s.: no significance (*n*=12). Genotypes: *AkhR-Gal4; UAS-CREB^RNAi^* or *AkhR-Gal4*. (F) cAMP levels in the brain were monitored using a genetically encoded Epac-camps sensor. Each data point represents one neuron, with at least five brains quantified per condition, *t*-test for statistical analysis. *P<0.05. Genotypes: *AkhR-Gal4; UAS-Epac-camps*.

To identify the neural substrates underlying CREB-dependent sleep regulation, we first examined the expression pattern of CREB in the brain upon starvation. We used 5XCRE-mCherry, a fluorescent reporter of CREB activity that drives expression of a destabilized mCherry (fused to a PEST sequence) under the control of five tandem CRE sites. *Ex vivo* imaging indicated a global increase in mCherry fluorescence in the brain after starvation, especially in the mushroom body (MB) and the subesophageal ganglion (SOG) (Fig. 3A,B), a key center for gustatory processing and feeding behavior (Hartenstein et al., 2018). Starvation induces the production of the glucagon-like peptide AKH from CCA, which in turn promotes the production of OA in AkhR-positive neurons within SOG (Fig. 3C) (Sun et al., 2023). Intriguingly, *CREB* knockdown in MB by OK107-Gal4 significantly reversed starvation-induced sleep loss (Fig. 3D). Similarly, silencing *CREB* in AkhR-expressing neurons by *AkhR*-GAL4 also remarkably alleviated starvation-associated sleep loss (Fig. 3E).

Epac1-camps is a FRET-based reporter for cAMP activity (Lissandron et al., 2007). Consistent with elevated CREB signaling, exchange protein directly activated by cAMP 1 (EPAC) activity was significantly increased in *Tdc2*-expressing neurons under starvation conditions (Fig. 3F). Together, these results indicate that CREB activation in AkhR-positive neurons is essential for starvation-induced sleep deprivation.

### CREB promotes OA biosynthesis by transcriptionally upregulating the expression of TβH

**Fig. 4.**
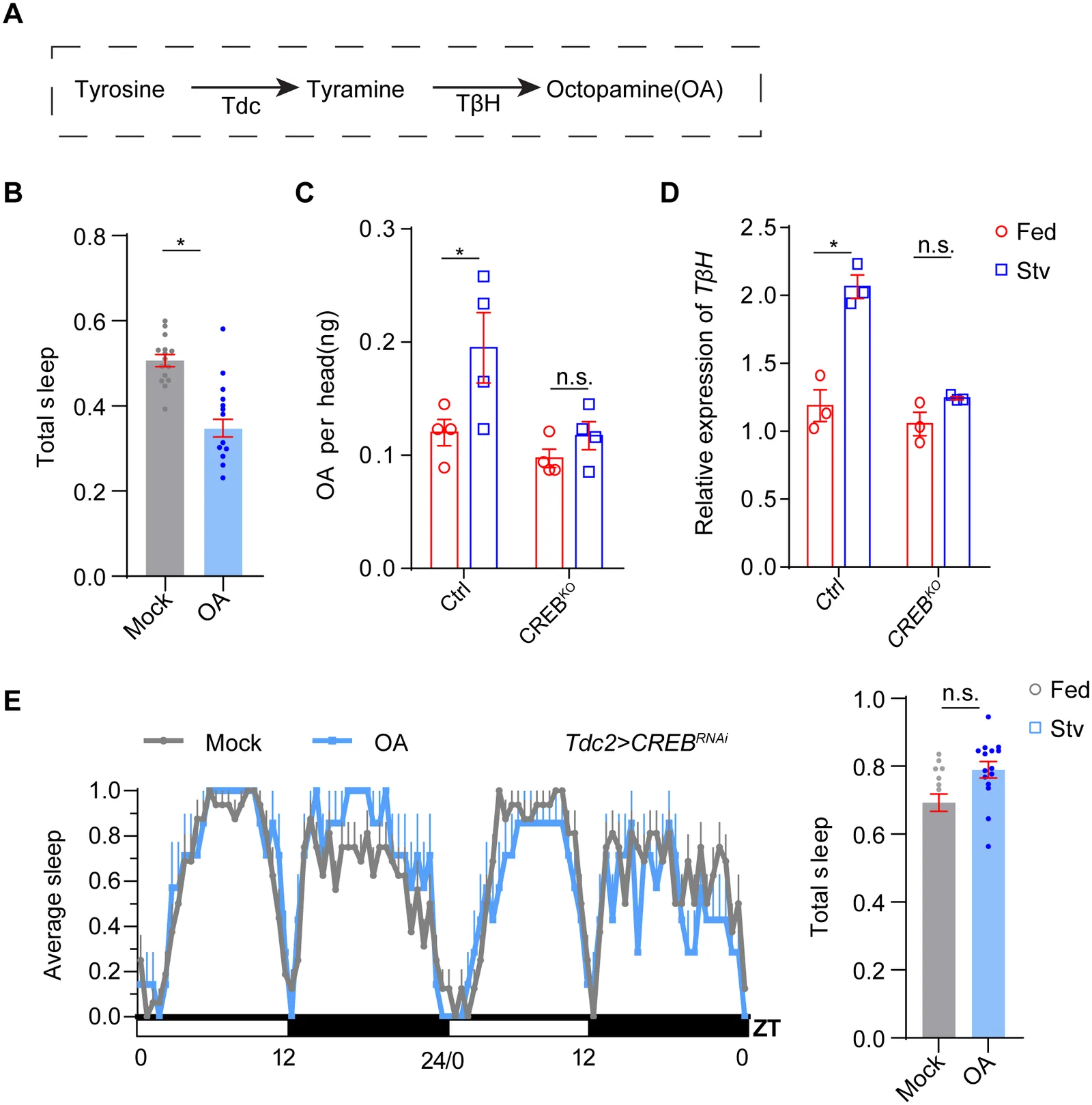
**CREB promotes OA biosynthesis by transcriptionally upregulating the expression of TβH.** (A) Tyramine is synthesized from tyrosine by tyrosine-decarboxylase (Tdc2). Tyramine can then be further processed into OA by TβH. (B) Total sleep was measured following a 24-h feeding period with OA-supplemented food (1 mg/ml). (C) OA level in fly heads of the indicated genotypes was quantified using HPLC analysis. Forty animals per group from four independent groups were analyzed. Mean±s.e.m. are shown, one-way ANOVA for statistical analysis. *P<0.05, n.s.: no significance (*n*=12). Genotypes: *w^1118^* or *CREB^Δ36^*. (D) Relative expression of TβH in the fly heads was examined by quantitative real-time PCR. Three independent groups were analyzed. Mean±s.e.m. are shown, two-way ANOVA for statistical analysis. *P<0.05, n.s.: no significance (*n*=12). Genotypes: *w^1118^* or *CREB^Δ36^*. (E) OA-promoted sleep was abolished when CREB was inhibited specifically in Tdc2-positive neurons. Representative sleep profiles are shown on the left, and quantification of total sleep amount is shown on the right (*n*=12). Mean±s.e.m. are shown. n.s.: no significance. Genotypes: *Tdc2-Gal4; UAS-CREB^RNAi^*.

In *Tdc2*-expressing neurons, tyramine is synthesized from tyrosine via tyrosine decarboxylase (TDC2) (Roeder, 2005) and is subsequently converted to OA by TβH (Monastirioti et al., 1996; Zhou et al., 2008) (Fig. 4A). Consistent with a previous study, dietary OA supplementation significantly increased locomotor activity and reduced sleep (Fig. 4B; Fig. S2A) (Yu et al., 2016; Yang et al., 2015). To determine whether CREB regulates OA production during starvation, we measured OA levels in fly heads using high-performance liquid chromatography (HPLC) (Fig. 4C). The results demonstrated that starvation significantly elevated OA levels, whereas this increase was abolished in a *CREB* null mutant, *CREB^Δ36^* (Ma et al., 2024) (Fig. 4C). Quantitative real-time PCR analysis further revealed that starvation specifically increased TβH mRNA expression, while *Tdc2* levels remained unchanged. Notably, this starvation-induced upregulation of TβH was completely lost in the *CREB^Δ36^* background (Fig. 4D; Fig. S2B).

*Tdc2*-Gal4 labels a subset of octopaminergic neurons in the SOG region (Zhou et al., 2008). However, cell-specific knockdown of *CREB* in *Tdc2*-positive neurons significantly suppressed sleep deprivation induced by exogenous OA (Fig. 4E; Fig. S2C).

Collectively, these findings support that CREB regulates sleep homeostasis by controlling OA production during starvation.

### Sleep dysfunction in a *Drosophila* HD model was partially rescued by *CREB* loss of function

**Fig. 5.**
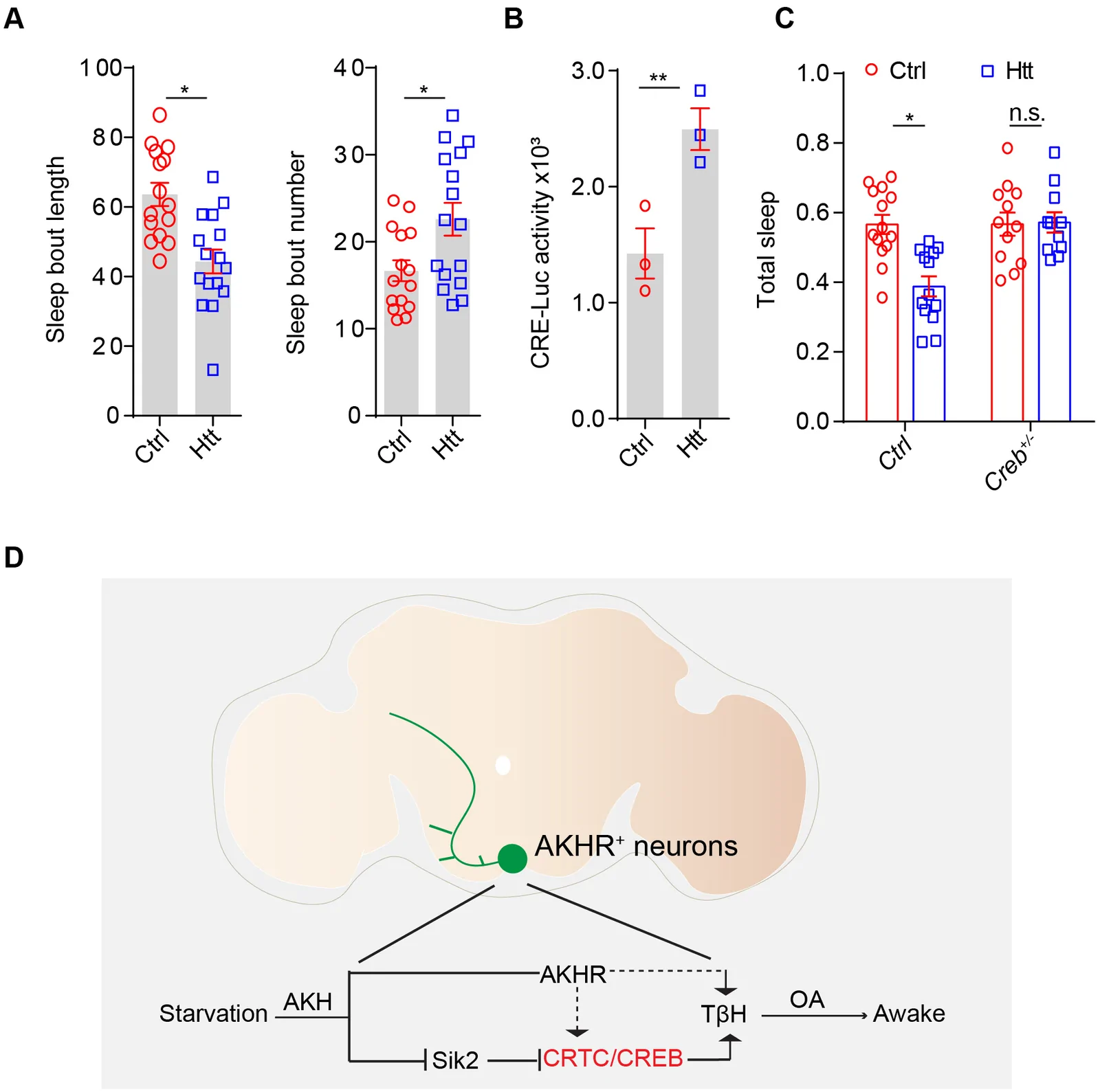
**Sleep failure associated with HD fly model was partially rescued in the CREB loss-of-function condition.** (A) Sleep bout length and number were analyzed following neuronal overexpression of Htt in the fly brain. Each dot represents an animal (*n*=16). Mean±s.e.m. are shown, *t*-test for statistical analysis. *P<0.05. (B) CRE-Luc reporter activity in the brain was compared between control and Htt-overexpressing groups. Biological triplicates were performed. Mean±s.e.m. were shown, *t*-test for statistical analysis. **P<0.01. (C) Total sleep over a 24-h period was compared between different genotypes. Each dot represents an animal (*n*=16). Mean±s.e.m. are shown, two-way ANOVA for statistical analysis. *P<0.05, n.s.: no significance. (D) Scheme: during starvation, CREB-mediated expression of TβH upregulates OA production. Concurrently, starvation promotes the expression of AKH, which acts on AkhR-positive neurons. In these neurons, AKHR signaling activates CREB via the SIK2/CRTC module in response to starvation. Genotypes for A and B: *Elav-GS-Gal4; UAS-HTT.ex1.Q120*. Genotypes for C: *Elav-GS-Gal4; UAS-HTT.ex1.Q120* or *Elav-GS-Gal4; UAS-HTT.ex1.Q120, CREB^Δ36^/+*.

Sleep disturbances and altered sleep architecture are common in HD patients (Arnulf et al., 2008). As reported previously (Gonzales and Yin, 2010), pan-neuronal expression of an exon 1 fragment of Htt with 120 polyglutamine repeats (UAS-HTT.ex1.Q120) in flies recapitulated HD-like sleep fragmentation, characterized by an increased number of sleep bouts and reduced bout length (Fig. 5A). Total sleep time was also altered during both day and night, resembling the perturbation pattern induced by starvation (Fig. S3). Intriguingly, CREB activity was also significantly elevated in *Elav*-GS>UAS-HTT.ex1.Q120 animals compared to controls (Fig. 5B).

Given the concurrent sleep disruption and elevated CREB activity, we then tested whether genetically reducing CREB activity could ameliorate the sleep phenotype. Indeed, Htt-induced sleep dysfunction was partially rescued in a CREB heterozygous mutant background (Fig. 5C; Fig. S3). These results demonstrated that CREB activity is involved in Htt-induced sleep fragmentation.

## DISCUSSION

Starvation elevates locomotion in animals, a behavioral response thought to mimic foraging and promote food acquisition (Yu et al., 2016). Although sleep-promoting and foraging-promoting neurons generally comprise distinct populations, they are functionally interconnected through antagonistic arousal pathways. During energy deficit, hunger signals – such as Akh in flies or ghrelin in mammals – activate central arousal systems. These systems, including OA neurons in *Drosophila* and orexin neurons in mammals (Roeder, 2020; Crocker et al., 2010), subsequently inhibit key sleep centers, including the dorsal-fan-shaped body in flies and the ventrolateral preoptic area in mammals, to suppress sleep and facilitate foraging behavior (Ma and Dan, 2025; Shafer and Keene, 2021). However, the neuronal circuits that orchestrate the dynamic control of sleep involving electrical activity patterns, neurochemical signaling, and synaptic plasticity are not fully understood.

Here, we show that starvation activates CREB in multiple brain regions, including the MB and the SOGs. Kenyon cells in the MB express OA receptors and are critical for sleep regulation (Reyes et al., 2026). Octopaminergic neurons are known to target the calyx of the MB. Following integration and processing within the MB, these signals dynamically regulate sleep behavior by acting on distinct MB output neurons (Zhao et al., 2026; Suárez-Grimalt et al., 2024). It would be intriguing to test whether elevated CREB in the MB also participates in sleep regulation during starvation.

On the other hand, recent studies showed that AKH released from the CCA activates AkhR-positive neurons, leading to increased OA production and the induction of locomotor hyperactivity. We further demonstrated that TβH, a rate-limiting enzyme for OA synthesis, is transcriptionally upregulated by CREB in response to starvation via the SIK2/CRTC cascade (Fig. 5D). These findings establish a molecular link between metabolic state and sleep-arousal regulation. However, exogenous OA-induced sleep deprivation remains elevated even when *CREB* was inhibited in octopaminergic neurons*.* One possibility is that the levels of other neurotransmitter(s) or neuropeptide(s) – such as glutamate (Chaverra et al., 2026), which is also an important sleep modulator in *Drosophila* – was altered by CREB to block OA-mediated sleep loss.

Future studies will be required to define this signaling cascade in greater detail and to explore potential crosstalk with other pathways mediated by different neuropeptides or neurotransmitters.

Chronic sleep deficiency is furthermore associated with an increased risk of serious health conditions, including metabolic disorders, cardiovascular disease, and depression. Here, we found that CREB was activated in neurons of a fly HD model and that CREB partial loss of function can alleviate multiple aspects of sleep disturbance, including fragmented, reduced sleep and nighttime hyperactivity. In addition to HD, sleep-related symptoms are widely recognized as core features of several neurodegenerative disorders, especially Alzheimer's disease and Parkinson's disease (Simmonds et al., 2025; Husain, 2021). It would be intriguing to explore whether manipulating CREB activity can also alleviate sleep abnormalities in these conditions.

We demonstrated that CREB within the foraging/arousal circuit can modulate its own output (via TβH regulation), creating a feedback loop that fine-tunes the trade-off between sleep pressure and foraging drive. These results provide a novel strategy for sleep failure associated with neurodegenerative diseases.

## MATERIALS AND METHODS

### *Drosophila* stocks and husbandry

*w^1118^* (3605), *5xCRE-Luc* (79016), *5xCRE-mCherry.PEST* (79020), *CREB^Δ36^* (79018), *UAS-CREB^RNAi^* (29332), *UAS-SIK2^OE^* (79023), *UAS-CRTC^RNAI^* (28886), *UAS-CBP^RNAI^* (31728), *UAS-Epac1-camps* (25409), *UAS-HTT.ex1.Q120* (68408) and *Elav-GS-GAL4* (43642) were obtained from Bloomington *Drosophila* Stock Center. Flies were maintained on standard molasses/yeast food at 25°C and 60% humidity under an 8AM:8PM light/dark cycle, unless otherwise specified.

### GeneSwitch-mediated transgene expression

The flies were administered RU486 (1 mg/ml, mifepristone; Calbiochem #475838) for 2 days to activate the Gal4 driver, after which they were transferred to testing tubes for sleep monitoring for another 2 days, with RU486 still present in the tubes.

### Luciferase assays

CRE-driven luciferase activity was measured using the Steady-Glo Luciferase Assay Kit (Promega, cat. #E2510). Freshly homogenized whole flies or dissected tissues (in 100 µl of Glo Lysis Buffer) were centrifuged at 12,000 ***g*** for 10 min. 30 µl of the supernatant was dispensed in triplicate into a 96-well plate, with three independent biological replicates per condition. After a 1-min dark incubation, luminescence was recorded on a Synergy HTX microplate reader (BioTek). Luciferase activity was normalized to total protein concentration as determined by a bicinchoninic acid (BCA) assay (YEASEN, cat. #20201ES76) using BSA standards.

### Measurement of OA levels in *Drosophila* heads by HPLC

OA levels in the brain were measured following a previously established protocol (Hardie and Hirsh, 2006). Briefly, adult *Drosophila* heads were freshly dissected and immediately homogenized in citrate-acetate buffer (pH 4.5). Homogenates were filtered and analyzed using an HPLC system equipped with a Jasco model PU-2080 Plus isocratic pump (Jasco Inc., Easton, MD), a Jasco model AS-950 Intelligent Autosampler, and an Antec Leyden Intro electrochemical detector (Antec Leyden, the Netherlands). OA standards were run at a concentration of 10 ng/Ml, and quantification was performed using two to three independent whole-head samples.

### Sleep monitoring and analysis using the *Drosophila* activity monitor (DAM) system

Flies of 5 to 10 days old were collected and loaded into glass behavioral tubes containing food of 5% sucrose and 2% agar. Tubes were placed in DAM (TriKinetics Inc., Waltham, MA, USA) and incubated at 25°C unless otherwise indicated. For starvation-related sleep assay, flies were first starved for 24 h on water-soaked Whatman paper. They were then transferred to test tubes containing only agar. Control flies were maintained on standard food throughout the corresponding period. Locomotion and sleep were analyzed by the 2020 version of the Sleep and Circadian Analysis MATLAB Program (SCAMP). In brief, the average sleep ratio was calculated using the *Drosophila* Activity Monitoring System as the mean of individual sleep ratios across all flies in the group, where the individual sleep ratio is the percentage of total sleep time in a period divided by the total monitoring time (Cichewicz and Hirsh, 2018).

### Quantitative real-time PCR

RNA was extracted from 15-20 guts using a TRIzol reagent (cat. #9109, Takara). cDNA was synthesized using an All-In-One 5X RT MasterMix Kit (cat. #G592, Applied Biological Materials) according to the manufacturer's instructions. Real-time PCR was performed in triplicate for each sample using a CFX96TM Real-Time System (Bio-Rad). Expression values were calculated using the ΔΔCt method and normalized to *Rp49* expression levels. Results are shown as mean±s.e.m. of at least three independent biological samples.

The following primer pairs were used:

Primers for TβH

F: CGGGCCGACAAGATACTTATAC

R: CGACTGCTTCTCCGGATTATT

Primers for *Tdc2*

F: GGTGGAGTACATCTGCAACTATC

R: CTTGGAGCAGTAGGCCATAAG

Primers for *RP49*

F: 5′-CCAGTCGGATCGATATGCTAAG-3′

R: 5′-CCGATGTTGGGCATCAGATA-3′.

### *Ex vivo Drosophila* intestine imaging setup

The imaging setup was based on our previous publications (Ma et al., 2024; Wang et al., 2025) with minor modifications. Flies were dissected in adult hemolymph-like saline (AHLS) culture medium containing 2 mM CaCl_2_, 5 mM KCl, 5 mM HEPES, 8.2 mM MgCl_2_, 108 mM NaCl, 4 mM NaHCO_3_, 1 mM NaH_2_PO_4_, 5 mM trehalose and 10 mM sucrose. Guts were immediately transferred into 35 mm Nunc™ glass-bottom dishes (Thermo Fisher Scientific™, 150682), embedded in 1% low-melting agarose (in AHLS) and immersed in AHLS. For all experiments, guts were fully embedded during live imaging.

### Neuronal cAMP level in the brain

To assess neuronal cAMP levels, brains were freshly dissected in AHLS medium and imaged *ex vivo* with a confocal microscopy. The relative YFP/cyan fluorescent protein (CFP) emission ratio of the Epac1-camps sensor was quantified from at least 20 cells per five animals for each experimental condition.

### Statistical analysis

We performed normality tests using the Shapiro–Wilk test prior to statistical analysis. All datasets presented in the manuscript have passed the normality tests. Data that passed normality tests (Shapiro–Wilk test) were plotted and analyzed using GraphPad Prism 8 and reported in the figure legends. Experimental flies and genetic controls were tested under the same conditions. All experiments were performed with at least three independent biological replicates. Statistical details are provided in the figure legends.

## Supplementary Material



10.1242/biolopen.062570_sup1 Supplementary information
