# Supplementary material for: CREB promotes starvation-induced sleep loss by regulating octopamine production in Drosophila
Source: Biol Open. 2026 Aug 21;15(8):bio062570. doi: 10.1242/bio.062570 (PMC13552482; doi:10.1242/bio.062570)
Supplement: Supplementary file 1 [file biolopen-15-062570-s1.pdf]

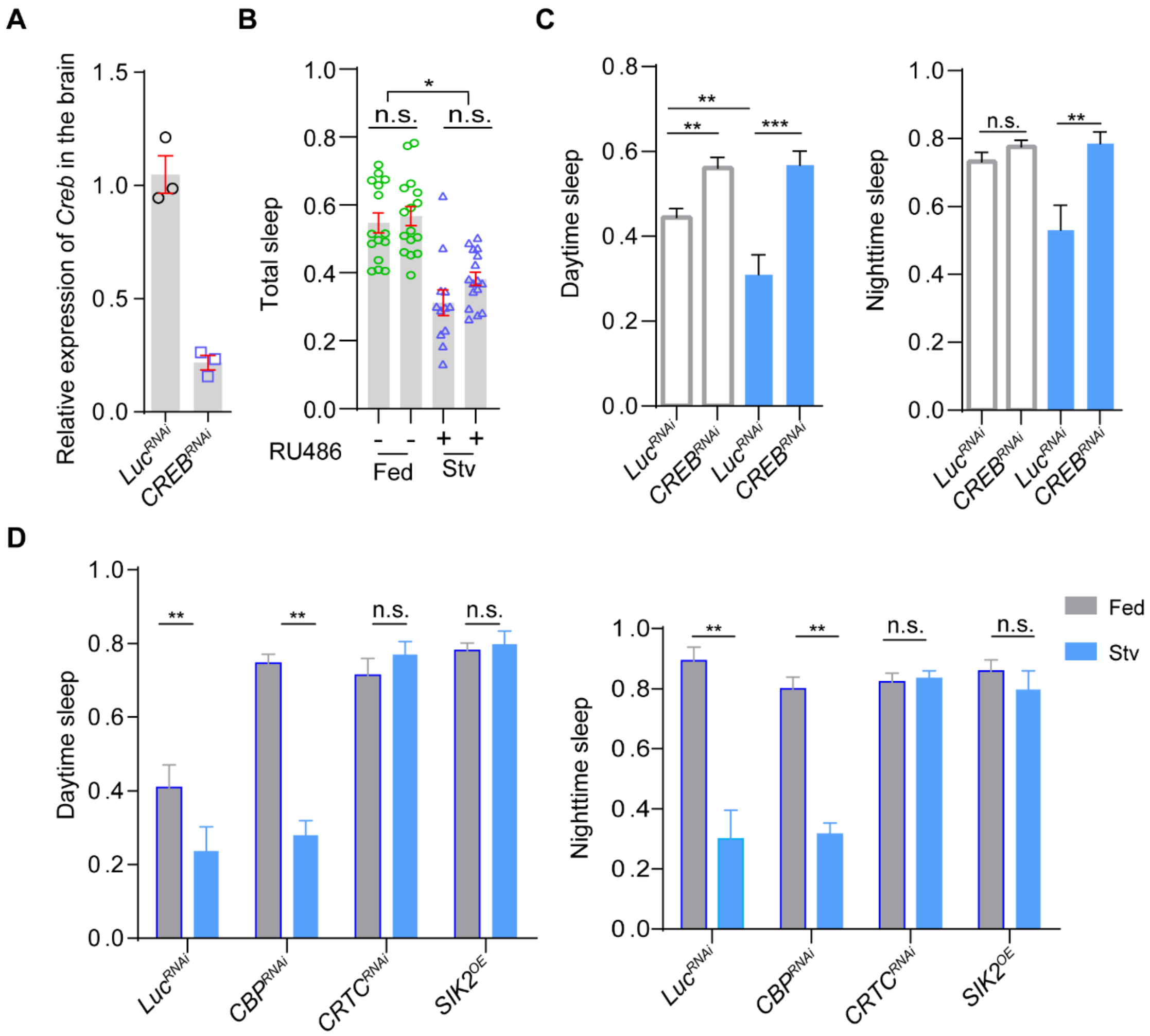

Fig. S1. related to **Fig.2** The SIK2/ CRTC module is required for starvation induced sleep deprivation.

A, RT-qPCR was used to quantify the relative expression level of *CREB* after its neuronal-specific silencing by *Elav-GS-Gal4*.

B, Total sleep was examined in *Elav-GS-Gal4* flies upon RU486-mediated induction under fed versus starved conditions.

C-D, Daytime and nighttime sleep patterns were quantified following the genetic manipulation of *CREB* and its upstream regulators. Mean± SEM are shown, one-way ANOVA for **B-C** and t-Test for **D**. **: p<0.01, ***: p<0.001, n.s.: no significance.

Genotypes for **A, C**: *Elav-GS-Gal4; UAS-Luc^{RNAi}* or *UAS-CREB^{RNAi}*, or *Elav-GS-Gal4; UAS-CREB^{RNAi}*. For **B**, *Elav-GS-Gal4,* for **D**: *Elav-GS-Gal4; UAS-Luc^{RNAi}* or *Elav-GS-Gal4; UAS-Cbp^{RNAi}*, or *Elav-GS-Gal4; UAS-Crtc^{RNAi}* or *Elav-GS-Gal4; UAS-SIK2^{OE}*.

Biology Open • Supplementary information

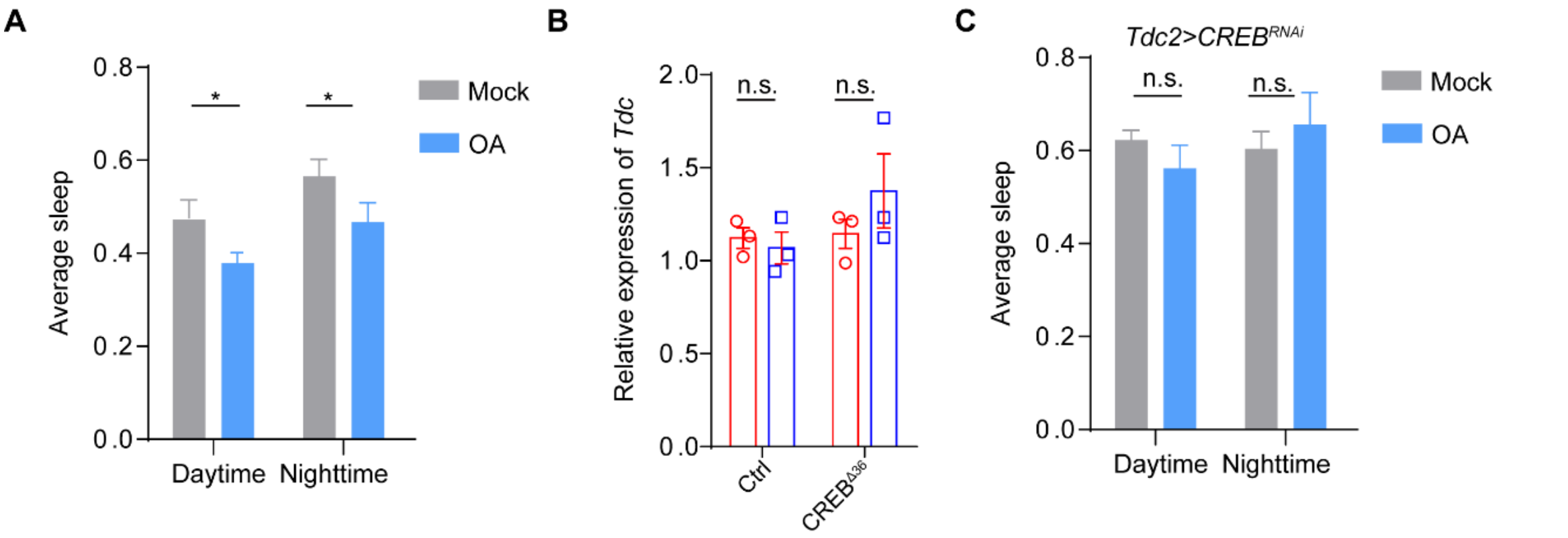

Fig. S2. Related to **Fig. 4** CREB is required for OA-mediated sleep suppression.

A, Average sleep quantity in the light and dark phases was measured in response to dietary OA supplementation. One way ANOVA for statistical analysis.
B, The relative expression of *Tdc* in the fly brain was assessed via RT-qPCR.
C, Average sleep quantity in the light and dark phases was measured with indicated genotypes. For all panels, mean± SEM are shown, one-way ANOVA for statistical analysis. *: $p<0.05$, n.s.: no significance.

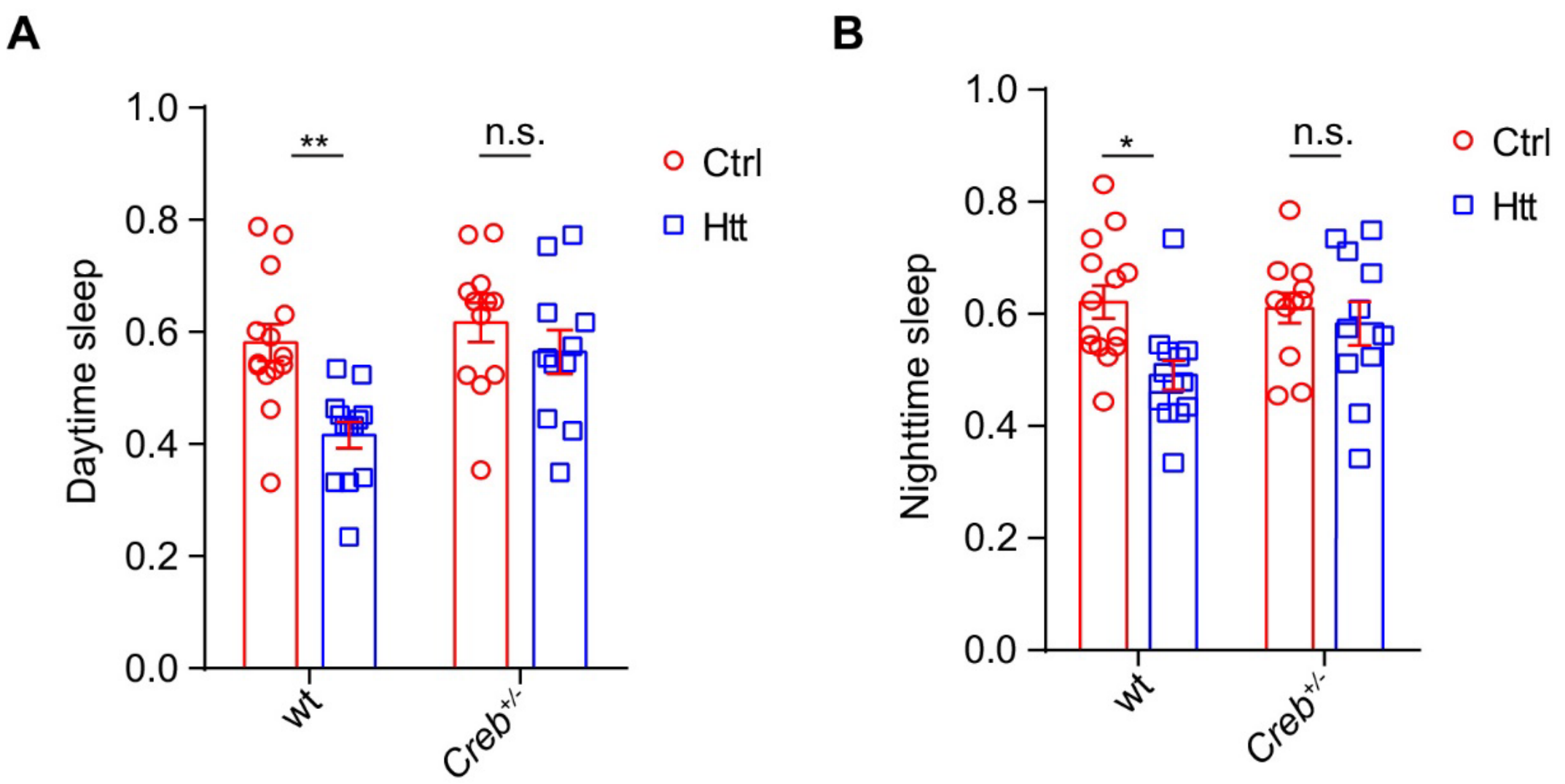

Fig. S3. Related to **Fig. 5** HTT-induced sleep deficits were partially suppressed by *CREB* loss of function.

A-B, Average sleep rate at daytime and nighttime was quantified with indicated genotypes. Two-way ANOVA for statistical analysis. *: $p<0.05$ **: $p<0.01$, n.s.: no significance. *Elav-GS-Gal4; UAS-HTT.ex1.Q120 or Elav-GS-Gal4; UAS-HTT.ex1.Q120, CREB*$^{\Delta 36}$/-.

Biology Open • Supplementary information

Original WB for **Fig.1G**

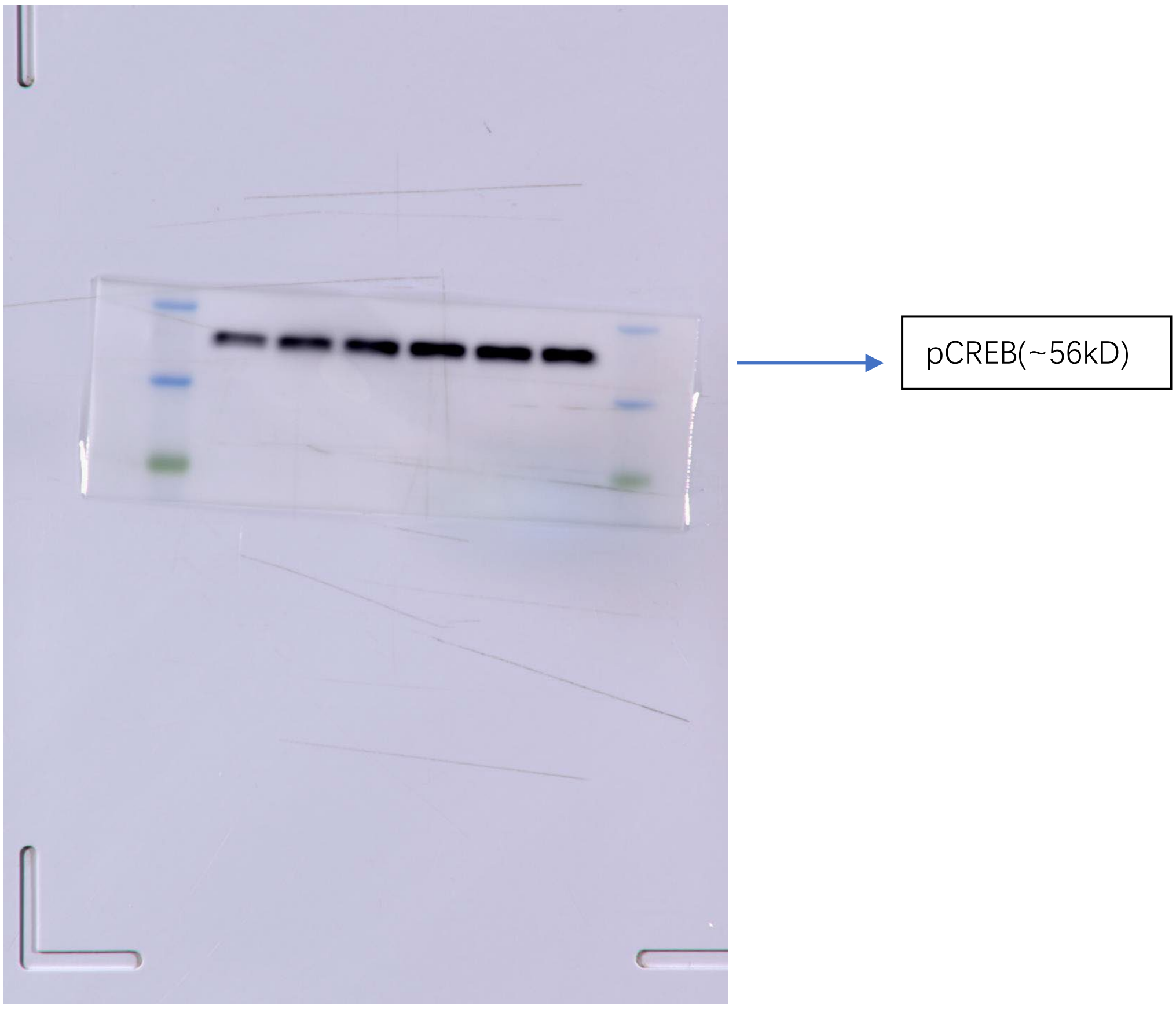

---ND--- ---Stv---

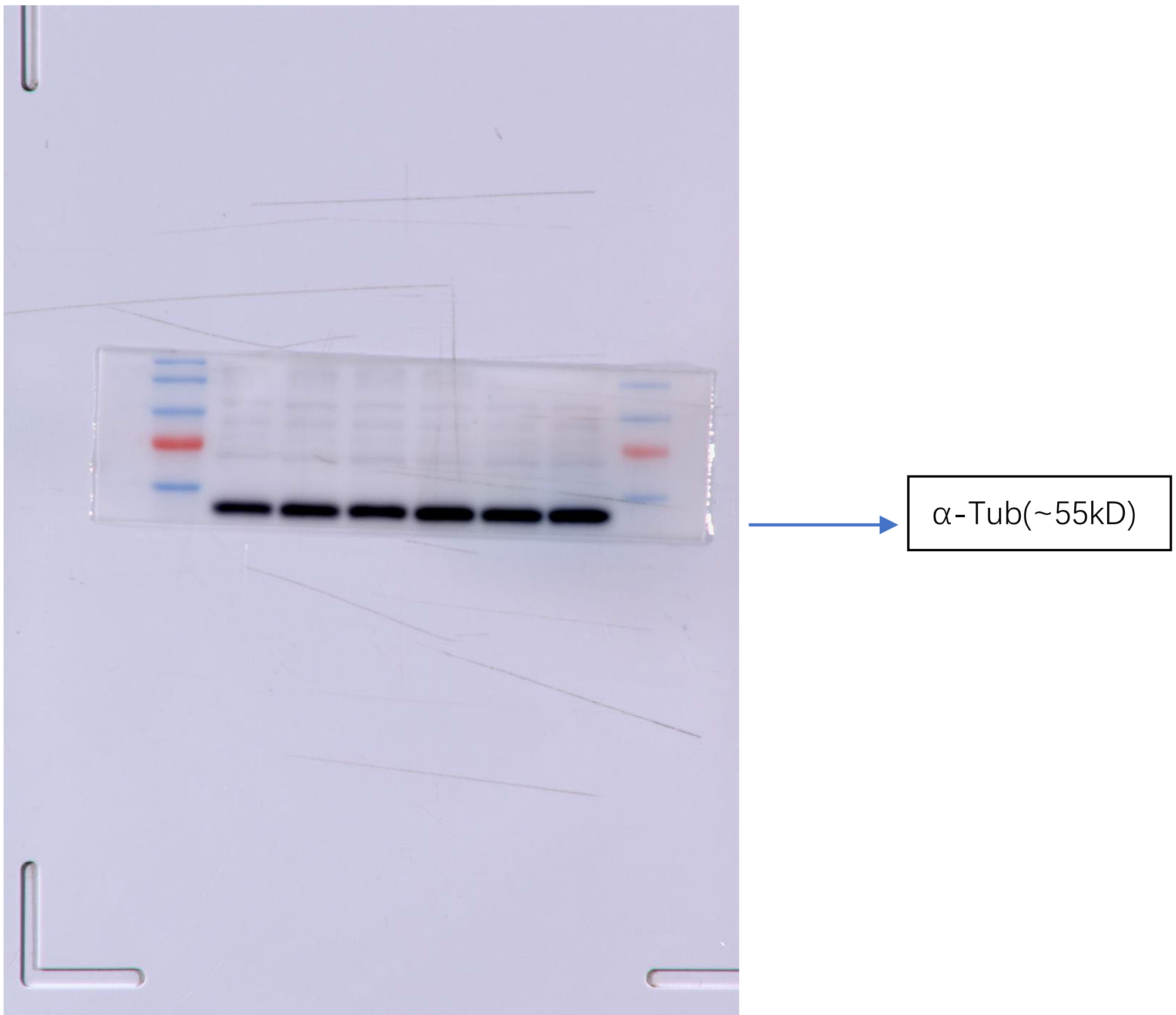

Biology Open • Supplementary information

Raw data for Fig.3F

Fed	Stv
0.4357818	0.5587328
0.3119243	0.5419244
0.3138649	0.4623861
0.3129052	0.3829005
0.3216744	0.4311614
0.2601363	0.3801343
0.2544455	0.3860821
0.2062996	0.3844445
0.2111458	0.3862919
0.1509031	0.3914528
0.2177383	0.3449031
0.2164972	0.3338314
0.2178213	0.3397712
0.1566316	0.3321563
0.1460518	0.3331527
0.1634501	0.2551116
0.1634508	0.2450889
0.2545321	0.2519244
0.2404519	0.2238692
0.2604508	0.2329055
0.2001332	0.2466734
0.2640812	0.2301363
0.2244454	0.2160821
0.2362991	0.2104445
	0.1429023

Normality and lognormality Test(GraphPad Prism 10)

Shapiro-Wilk test

W 0.9227 0.9477

P value 0.0669 0.2221

Passed normality test (alpha=0.05)? Yes Yes

P value summary ns ns

Kolmogorov-Smirnov test(GraphPad Prism 10)

KS distance 0.1471 0.1622

P value >0.1000 0.0886

Passed normality test (alpha=0.05)? Yes Yes

P value summary ns ns

Biology Open • Supplementary information
