# Supplementary material for: CREB promotes starvation-induced sleep loss by regulating octopamine production in Drosophila
Source: Biol Open. 2026 Aug 21;15(8):bio062570. doi: 10.1242/bio.062570 (PMC13552482; doi:10.1242/bio.062570)
Supplement: Supplementary file 2 [file biolopen-15-062570_review_history.pdf]

CREB promotes starvation-induced sleep loss by regulating octopamine production in Drosophila

Shiye Zhu, Zhian Rao, Youjie Yin, Shuxin Chen and Hansong Deng
10.1242/bio.062570

Editor: Sandhya Koushika

Review timeline

Original submission:	26 March 2026
Editorial decision:	3 April 2026
First revision received:	19 June 2026
Editorial decision:	25 June 2026
Second revision received:	28 June 2026
Accepted:	14 July 2026

Original submission

First decision letter

MS ID#: bio.062570

MS Title: CREB promotes starvation-induced sleep loss by regulating octopamine production in Drosophila

Authors: Shiye Zhu, Zhian Rao, Youjie Yin, Shuxin Chen and Hansong Deng

I have now reached a decision on the above manuscript.

The reviewer reports are shown at the bottom of this email.

As you will see, the reviewers raised a number of substantial criticisms that prevent me from accepting the paper at this stage.

They suggest, however, that a revised version might prove acceptable, if you can address their concerns. If you think that you can deal satisfactorily with the criticisms on revision, I would be pleased to see a revised manuscript. We would then return it to the reviewers.

At this stage, we also ask you to ensure your manuscript complies with our formatting guidelines. Provided you are able to fully address the referees' comments, we are positive about publication of your paper (we accept over 95% of revision submissions) and therefore hope you won't mind any extra work involved in reformatting your manuscript at this point.

Please upload both a 'clean' version of your Word file, along with a highlighted version clearly showing where you have made changes in the revised manuscript. Please avoid using 'Track changes' in Word files as these are lost in PDF conversion.

I should be grateful if you would also provide a point-by-point response detailing how you have dealt with the points raised by the reviewers in the 'Response to Reviewers' box. Please attend to

© 2026. Published by The Company of Biologists under the terms of the Creative Commons Attribution License (https://creativecommons.org/licenses/by/4.0/).

all of the reviewers' comments. If you do not agree with any of their criticisms or suggestions please explain clearly why this is so.

Reviewer 1

Comments for the author

This paper identifies CREB as a key transcriptional regulator linking metabolic state to sleep behaviour in Drosophila. During food deprivation, the SIK2/CRTC pathway activates CREB in octopaminergic neurons, driving upregulation of TβH and consequently increased octopamine production, which suppresses sleep. Notably, reducing CREB activity also ameliorates sleep disruption in a Huntington's disease fly model, pointing to CREB hyperactivation as a shared mechanism across metabolically- and neurodegeneration-driven sleep pathologies.
While the approach is valuable and the findings are important and fitting to the journal's scope, I have several points of concern regarding some experimental controls, methods, and conclusions that I would like to see addressed in order to recommend publication of this manuscript.

Major points

1) The ddH2O-based starvation method is not described. Given the centrality of this method to this paper, the authors should detail what it entails and the time courses of the various measurements in relation to the starvation period. In particular, during sleep recordings, were "starved" flies loaded in tubes equipped with sugar food, like their "fed" controls? Can the authors comment on this possible confounder?

2) To maintain their claims, authors should provide a fed-control for the experiment shown in Figure 3A.

3) The authors show that "Cell-specific knockdown of CREB in Tdc2-positive neurons significantly suppressed sleep deprivation induced by exogenous OA". Given the model illustrated in Figure 5D, wouldn't the authors expect that exogenous administration of OA would overcome any interference with manipulations of the upstream release circuitry? Are there possibly other pathways involved? Could the authors comment on this?

4) The CREB null mutant is introduced suddenly in Figure 4C, D, and its use is not justified as opposed to the RNAi-mediated knockdown for conceptually similar experiments in the remainder of the paper. Can the authors repeat those experiments with the CREBRNAi they used elsewhere?

5) In the statistics section, authors observe "Data that passed normality tests (Shapiro-Milk test) were plotted and analysed?" What happened to the data that did not pass the test? Do the authors refer to entire datasets or replicates? What's the reason for excluding non-normal data?

Minor points:

1) Sleep traces in Figure 2a should include error bars.

2) The authors write "The Drosophila genome encodes the majority of neurotransmitter systems involved in sleep and circadian rhythm(3-5)". As it stands, the sentence is pointless, since obviously, the fly displays sleep and circadian rhythms, and their neuromodulators. Maybe the authors want to refer to similarities between those and the mammalian ones?

3) What is "average sleep ratio"? Authors should explain it in the methods. There are also some inconsistencies with "Average sleep" and "total sleep" labels.

4) In Fig.1A, the sample size is the same as b-d? If so, clarify it there and in the rest of the paper

5) Can the authors comment on their findings of the involvement of the mushroom body? Can they possibly relate them to the findings included in the preprint Reyes et al, 2025, bioRxiv

© 2026. Published by The Company of Biologists under the terms of the Creative Commons Attribution License (https://creativecommons.org/licenses/by/4.0/).

6) After how many days from the RU administration is sleep recorded? The authors say two days, but are those two days the ones shown in the sleep recordings?

7) The colour scheme in Figure 2b-c could be clarified better

8) Can the authors comment on the lack of rescue effect with the CBPRNAi?

Reviewer 2

Comments for the author

This manuscript is generally quite clear, and I very much appreciate the succinctness of the results. However, I have a few concerns which led to me selecting "no" for completeness and reproducibility:

The units on Figure 1E-F are not entirely clear to me. I understand it is somehow normalized to total protein content, but that doesn't clear it up. More clear labeling and/or references in the methods would fix this.

In the experiment using the drug-inducible Elav-GS-GAL4 driven CREB knockdown: (i) Were the two control conditions also fed the drug? (ii) Elav>Luc-RNAi seem the appropriate control, but I see the authors also tested the UAS-CREB-RNAi parent line alone; any particular reason the GAL4 parent line wasn't tested alone?

Figure 3, Lines 171-180: Could the authors show a representative images from fed and starved flies? Is there any quantification (this seems necessary to me)? I see no evidence of an increase in these data without some sort of comparison. I also don't see any region specificity, but it's difficult to know without quantification.

Figure 3E: why do the data appear almost quantized? i.e., they seem to cluster around certain values. It was curious to me that they are not continuously and normally distributed (although they may be the latter)

Minor: (i) Line 146 ", luciferase activity was significantly elevated in whole-fly extracts and in fly heads upon starvation and increased." - not sure what was meant by "increased"; (ii) Figure 1 A-F labels seem incorrect and out of order

Reviewer's Responses to Questions

Experimental quality
Does each figure have the proper controls?
If 'No', please indicate reasons in Comments for Author box below.
Reviewer #1:
- No

Reviewer #2:
- Yes

Were the data analyzed using appropriate statistical tests?
If 'No', please indicate reasons in Comments for Author box below.
Reviewer #1:
- Yes

Reviewer #2:
- Yes

© 2026. Published by The Company of Biologists under the terms of the Creative Commons Attribution License (https://creativecommons.org/licenses/by/4.0/).

Reproducibility
Were experiments performed using adequate number of biological replicates?
If 'No', please indicate reasons in Comments for Author box below.
Reviewer #1:
- Yes

Reviewer #2:
- Yes

Does the methods section provide sufficient detail to permit reproducibility?
If 'No', please indicate reasons in Comments for Author box below.
Reviewer #1:
- Yes

Reviewer #2:
- No

Completeness
Are the manuscript's conclusions supported by the data?
If 'No', please indicate reasons in Comments for Author box below.
Reviewer #1:
- No

Reviewer #2:
- No

Scholarship
Do the authors cite and discuss the merits of data that would argue for and against their conclusion?
If 'No', please indicate reasons in Comments for Author box below.
Reviewer #1:
- No

Reviewer #2:
- Yes

Does the manuscript title & abstract accurately reflect the contents of the manuscript, without hyperbole?
If 'No', please indicate reasons in Comments for Author box below.
Reviewer #1:
- Yes

Reviewer #2:
- Yes

First revision

Author response to reviewers' comments

We appreciate the time and effort that the editor and the reviewers have dedicated to our manuscript. The revised manuscript has been greatly improved based on the reviewers' comments. We have fully addressed all of the reviewers' concerns and provide below a point-by-point response:

© 2026. Published by The Company of Biologists under the terms of the Creative Commons Attribution License (https://creativecommons.org/licenses/by/4.0/).

Comments from the Reviewers:

Reviewer 1: This paper identifies CREB as a key transcriptional regulator linking metabolic state to sleep behaviour in Drosophila. During food deprivation, the SIK2/CRTC pathway activates CREB in octopaminergic neurons, driving upregulation of TβH and consequently increased octopamine production, which suppresses sleep. Notably, reducing CREB activity also ameliorates sleep disruption in a Huntington's disease fly model, pointing to CREB hyperactivation as a shared mechanism across metabolically- and neurodegeneration-driven sleep pathologies.
While the approach is valuable and the findings are important and fitting to the journal's scope, I have several points of concern regarding some experimental controls, methods, and conclusions that I would like to see addressed in order to recommend publication of this manuscript.
We thank the reviewer for the positive comments and valuable suggestions. The manuscript has been significantly improved by incorporating the reviewer's feedback.

Major points

1) The ddH2O-based starvation method is not described. Given the centrality of this method to this paper, the authors should detail what it entails and the time courses of the various measurements in relation to the starvation period. In particular, during sleep recordings, were "starved" flies loaded in tubes equipped with sugar food, like their "fed" controls? Can the authors comment on this possible confounder?
We agree with the reviewer that it is necessary to clarify it since it was utilized throughout the study.

To examine the effect of starvation during sleep, flies were starved on water-soaked Whatman paper for 24 hours, after which they were placed into testing tubes containing only agar. Control flies were provided with standard food throughout the period. Locomotion and sleep activity was then recorded over the subsequent days.

We have updated the method section (**Lines 121-114**) accordingly.

2) To maintain their claims, authors should provide a fed-control for the experiment shown in Figure 3A.
We agree, and the control panel has now been included in **Fig. 3A**. Quantifications are also shown in **newFig.3B**.

3) The authors show that "Cell-specific knockdown of CREB in Tdc2-positive neurons significantly suppressed sleep deprivation induced by exogenous OA". Given the model illustrated in Figure 5D, wouldn't the authors expect that exogenous administration of OA would overcome any interference with manipulations of the upstream release circuitry? Are there possibly other pathways involved? Could the authors comment on this?
We fully understand the reviewer's concern. It is indeed surprising that knockdown of CREB in Tdc2-positive neurons suppressed octopamine (OA)-associated sleep deprivation, given that OA is the end product of this pathway.
One possibility is that CREB in octopaminergic neurons could alter the levels of other neurotransmitter(s) or neuropeptide(s)—such as glutamate (PMID: 41511468), which is also an important sleep modulator in *Drosophila*—and this change would then block OA-mediated sleep loss. It would be intriguing to further elucidate the underlying mechanism in the future.

We have updated the discussion section in **lines 278-280** to clarify it.

4) The CREB null mutant is introduced suddenly in Figure 4C, D, and its use is not justified as opposed to the RNAi-mediated knockdown for conceptually similar experiments in the remainder of the paper. Can the authors repeat those experiments with the CREBRNAi they used elsewhere?

In Fig. 4C and 4D, we examined the expression levels of *TβH* and *Tdc2* in the brain. We chose the null mutant of *CREB*, which has been validated in our previous studies (PMC11329785, PMC9357022, PMC12966286), because neuron-specific knockdown of *CREB* would reduce CREB expression in only a subset of neurons. Therefore, the *CREB*-null mutant allows for more sensitive detection of changes

© 2026. Published by The Company of Biologists under the terms of the Creative Commons Attribution License (https://creativecommons.org/licenses/by/4.0/).

in the expression levels of these genes across the whole brain. Thus, we employed the null allele rather than RNAi in this experimental set.

5) In the statistics section, authors observe "Data that passed normality tests (Shapiro-Milk test) were plotted and analysed?" What happened to the data that did not pass the test? Do the authors refer to entire datasets or replicates? What's the reason for excluding non-normal data?

We apologize for the confusion. For all datasets, we performed normality tests using the Shapiro-Wilk test prior to statistical analysis. All datasets presented in the manuscript have passed the normality tests. We have updated the Methods section accordingly.

Minor points:

1) Sleep traces in Figure 2a should include error bars.
The error bars are included in the revised version.

2) The authors write "The Drosophila genome encodes the majority of neurotransmitter systems involved in sleep and circadian rhythm(3-5)". As it stands, the sentence is pointless, since obviously, the fly displays sleep and circadian rhythms, and their neuromodulators. Maybe the authors want to refer to similarities between those and the mammalian ones?
We indeed intended to highlight the similarity of Drosophila and mammals. We have rewritten the sentence by saying that "*The Drosophila genome contains counterparts of most mammalian neurotransmitter systems that regulate sleep and circadian rhythm*" in **Line 37**. We have also gone through the entire manuscript to check for grammatical errors and language issues. Thank you for pointing this out.

3) What is "average sleep ratio"? Authors should explain it in the methods. There are also some inconsistencies with "Average sleep" and "total sleep" labels.
In brief, the average sleep ratio was calculated using the Drosophila Activity Monitoring System (DAMS) as the mean of individual sleep ratios across all flies in the group, where the individual sleep ratio is the percentage of total sleep time in a period divided by the total monitoring time (PMC6002956). We have updated the method section(**line121-124**) accordingly.

4) In Fig.1A, the sample size is the same as b-d? If so, clarify it there and in the rest of the paper
Yes, and the sample sizes are now clarified throughout the paper, as suggested.

5) Can the authors comment on their findings of the involvement of the mushroom body? Can they possibly relate them to the findings included in the preprint Reyes et al, 2025, bioRxiv
As the reviewer pointed out, the mushroom body (MB) is also critical for sleep regulation. Octopamine is known to target neurons expressing octopamine receptors in the MB. Following integration and processing within the MB, these signals dynamically regulate sleep behavior by acting on distinct MB output neurons (MBONs) (PMC11199956, PMC12907755).
Consistently, the reference mentioned by the reviewer (iScience, 2026; PMCID: PMC13122709, cited as reference [45]) also indicates that neurons expressing octopamine receptors in the mushroom body mediate sleep behavior.
Since CREB activity is upregulated in the MB upon starvation, it would be intriguing to test whether CREB in the MB also involves in starvation-associated sleep loss. Dissecting the neuronal circuit underlying this process would be a valuable direction for future research. We have added these points to the Discussion section (**Lines 264 to 270**).

6) After how many days from the RU administration is sleep recorded? The authors say two days, but are those two days the ones shown in the sleep recordings?
Sorry for the confusion. The flies were administered RU486 for two days to activate the Gal4 driver, after which they were transferred to testing tubes for sleep monitoring for another two days, with RU486 still present in the tubes. We have updated the Methods section and the figure legends accordingly.

7) The colour scheme in Figure 2b-c could be clarified better
We have changed them as suggested.

© 2026. Published by The Company of Biologists under the terms of the Creative Commons Attribution License (https://creativecommons.org/licenses/by/4.0/).

8) Can the authors comment on the lack of rescue effect with the CBPRNAi?
CBP/p300 is a coactivator for multiple transcription factors, such as c-Fos and AP-1. It's possible that the effect of CBP may be complicated by the involvement of other transcription factors that regulate sleep.

Reviewer 2: This manuscript is generally quite clear, and I very much appreciate the succinctness of the results. However, I have a few concerns which led to me selecting "no" for completeness and reproducibility:
We thank the reviewer for their positive comments and valuable efforts to improve our manuscript.

The units on Figure 1E-F are not entirely clear to me. I understand it is somehow normalized to total protein content, but that doesn't clear it up. More clear labeling and/or references in the methods would fix this.
Thank you for pointing this out. The figure legends have been updated.

In the experiment using the drug-inducible Elav-GS-GAL4 driven CREB knockdown: (i) Were the two control conditions also fed the drug? (ii) Elav>Luc-RNAi seem the appropriate control, but I see the authors also tested the UAS-CREB-RNAi parent line alone; any particular reason the GAL4 parent line wasn't tested alone?
Sleep behavior is sensitive to genetic background. Therefore, as the reviewer noted, we included multiple genetic controls for our experimental datasets. We did not include the GAL4 parent line (elavGSGal4) because it has been validated for sleep assays in *Drosophila* in previous studies (DOI: 10.1126/science.aat1650).

Figure 3, Lines 171-180: Could the authors show a representative images from fed and starved flies? Is there any quantification (this seems necessary to me)? I see no evidence of an increase in these data without some sort of comparison. I also don't see any region specificity, but it's difficult to know without quantification.
Thank you for the suggestion. The control has now been included in **Fig.3A**. We fully agree with the reviewer that quantification is necessary for comparison. As quantified in new**Fig.3B**, significant more mCherry fluorescence level in mushroom body and SOGs region in brain after starvation compared with control.
Indeed, starvation induced a global increase in mCherry fluorescence in the brain, indicating CREB activation. These results are consistent with the CRE-Luc data in **Figs. 1E-F** and with our new western blot analysis, which we have now included as **Fig. 1G**. The reason we focus on MB and SOG regions in the brain because these two regions are relatively easy to identify and has been shown to regulate sleep in *Drosophila*.

Figure 3E: why do the data appear almost quantized? i.e., they seem to cluster around certain values. It was curious to me that they are not continuously and normally distributed (although they may be the latter)
All the datasets have passed normality tests before statistical analysis. As for Fig. 3E, used to measure cAMP levels by FRET assay, we have double-checked the original values, and the scattered distribution is likely due to rounding of decimal places.

Minor: (i) Line 146 ", luciferase activity was significantly elevated in whole-fly extracts and in fly heads upon starvation and increased." - not sure what was meant by "increased"; (ii) Figure 1 A-F labels seem incorrect and out of order
We apologize for the confusion. We have deleted the word "increased" and carefully gone through the manuscript to correct any grammatical errors. The panels have been updated accordingly. We would like to thank the reviewer once again for their valuable suggestions, which have significantly strengthened the manuscript.

<u>Second decision letter</u>

MS ID#: bio.062570R1

© 2026. Published by The Company of Biologists under the terms of the Creative Commons Attribution License (https://creativecommons.org/licenses/by/4.0/).

MS Title: CREB promotes starvation-induced sleep loss by regulating octopamine production in Drosophila

Authors: Shiye Zhu, Zhian Rao, Youjie Yin, Shuxin Chen and Hansong Deng

I have now reached a decision on the above manuscript.

The reviewer reports are shown at the bottom of this email.

As you will see, the reviewers gave favourable reports, but raised some critical points that will require amendments to your manuscript. I hope that you will be able to carry these out, because we would like to be able to accept your paper.

At this stage, we also ask you to ensure your manuscript complies with our formatting guidelines – please see our manuscript preparation guidelines for details. Provided you are able to fully address the referees' comments, we are positive about publication of your paper (we accept over 95% of revision submissions) and therefore hope you won't mind any extra work involved in reformatting your manuscript at this point.

Please upload both a 'clean' version of your Word file, along with a highlighted version clearly showing where you have made changes in the revised manuscript. Please avoid using 'Track changes' in Word files as these are lost in PDF conversion.

I should be grateful if you would also provide a point-by-point response detailing how you have dealt with the points raised by the reviewers in the 'Response to Reviewers' box. Please attend to all of the reviewers' comments. If you do not agree with any of their criticisms or suggestions please explain clearly why this is so.

Reviewer 1

Comments for the author

The authors have addressed my comments and the paper is even stronger now.

The only inconsistency that would be worth amending is the use of "ND" in Fig. 3 which is not defined and different from the rest of the paper. Maybe "Fed" is more appropriate?

Reviewer 2

Comments for the author

The authors state that "We did not include the GAL4 parent line (elavGSGal4) because it has been validated for sleep assays in Drosophila in previous studies." That elav-GS-GAL4 has been previously used does not seem sufficient justification to not use it here. I may be mistaken, but I do not believe the cited study compares starved vs fed elav-GS-GAL4 flies, so even if it has been previously validated, this study does not demonstrate that (please correct me if I'm wrong). To reiterate, the concern is exactly as the authors have themselves suggested: the parental GAL4 line could easily have some phenotype unrelated to CREB knockdown that may also be present in the F1. As pointed out in my original comment, the authors essentially demonstrate that this is probably not the case by using elav-GS-GAL4>UAS-Luc-RNAi as a control, so using the GAL4 alone is not strictly necessary. That said, any Drosophila biologist reader will question why it is missing - it would greatly strengthen their claims to include the elav-GS-GAL4 background control. Alternatively, the authors should cite the most relevant studies to inspire confidence that the parental GAL4 strain would not have an appreciable phenotype in this assay.

In response to the distribution of data in Figure 3E, I still would have expected these data to be continuous, and the authors' explanation that it is the result of rounding is not convincing without details of their rounding methodology. The authors should explain their rounding to ensure reproducibility, and/or include the raw data so that one can "assess the rigor of the analysis".

© 2026. Published by The Company of Biologists under the terms of the Creative Commons Attribution License (https://creativecommons.org/licenses/by/4.0/).

The authors seem to have made a copy-paste mistake. I believe they pasted a response to reviewers in the methods section "GeneSwitch-mediated transgene expression" (that section includes the phrase "We have updated the Methods section and the figure legends accordingly").

Reviewer's Responses to Questions

Experimental quality
Are experiments performed using appropriate methods that will answer the question (or test the hypothesis or support the observations) posed by the authors? Is the right tool used for the job?
If 'No', please indicate reasons in Comments for Author box below.
Reviewer #1:
- Yes

Reviewer #2:
- Yes

Were the data analyzed using appropriate statistical tests?
If 'No', please indicate reasons in Comments for Author box below.
Reviewer #1:
- Yes

Reviewer #2:
- Yes

Reproducibility
Were experiments performed using adequate number of biological replicates?
If 'No', please indicate reasons in Comments for Author box below.
Reviewer #1:
- Yes

Reviewer #2:
- Yes

Is there sufficient raw data to assess the rigor of the analysis?
If 'No', please indicate reasons in Comments for Author box below.
Reviewer #1:
- No

Reviewer #2:
- No

Does the methods section provide sufficient detail to permit reproducibility?
If 'No', please indicate reasons in Comments for Author box below.
Reviewer #1:
- Yes

Reviewer #2:
- No

Completeness
Are the manuscript's conclusions supported by the data?
If 'No', please indicate reasons in Comments for Author box below.
Reviewer #1:

© 2026. Published by The Company of Biologists under the terms of the Creative Commons Attribution License (https://creativecommons.org/licenses/by/4.0/).

- Yes

Reviewer #2:
- Yes

Scholarship
Do the authors cite and discuss the merits of data that would argue for and against their conclusion?
If 'No', please indicate reasons in Comments for Author box below.
Reviewer #1:
- Yes

Reviewer #2:
- Yes

Does the manuscript title & abstract accurately reflect the contents of the manuscript, without hyperbole?
If 'No', please indicate reasons in Comments for Author box below.
Reviewer #1:
- Yes

Reviewer #2:
- Yes

Second revision

Author response to reviewers' comments

Comments from the Reviewers:

Reviewer 1: The authors have addressed my comments and the paper is even stronger now.
We would like to express our sincere gratitude to the reviewer for the helpful feedback, which has greatly enhanced the quality of our manuscript.

The only inconsistency that would be worth amending is the use of "ND" in Fig. 3 which is not defined and different from the rest of the paper. Maybe "Fed" is more appropriate?
ND stands for normal diet. We fully agree and have changed them into "Fed" for consistency.

Reviewer 2: The authors state that "We did not include the GAL4 parent line (elavGSGal4) because it has been validated for sleep assays in Drosophila in previous studies." That elav-GS-GAL4 has been previously used does not seem sufficient justification to not use it here. I may be mistaken, but I do not believe the cited study compares starved vs fed elav-GS-GAL4 flies, so even if it has been previously validated, this study does not demonstrate that (please correct me if I'm wrong). To reiterate, the concern is exactly as the authors have themselves suggested: the parental GAL4 line could easily have some phenotype unrelated to CREB knockdown that may also be present in the F1. As pointed out in my original comment, the authors essentially demonstrate that this is probably not the case by using elav-GS-GAL4>UAS-Luc-RNAi as a control, so using the GAL4 alone is not strictly necessary. That said, any Drosophila biologist reader will question why it is missing - it would greatly strengthen their claims to include the elav-GS-GAL4 background control. Alternatively, the authors should cite the most relevant studies to inspire confidence that the parental GAL4 strain would not have an appreciable phenotype in this assay.
We are fully aware that proper genetic controls are key for these behavioral experiments. As acknowledged by the reviewer, *elav-GS-GAL4>UAS-Luc-RNAi* serves as an appropriate control for

© 2026. Published by The Company of Biologists under the terms of the Creative Commons Attribution License (https://creativecommons.org/licenses/by/4.0/).

elav-GS-GAL4>UAS-CREB-RNAi, as both lines use the same driver and carry the same number of transgene copies, thereby avoiding GAL4 titration effects.

Nevertheless, we agree with the reviewer that further characterization of the driver itself in response to starvation would be beneficial for the study. As shown in the new panel of **supplemental Fig.1B**, our new results showed that sleep behavior remains largely unchanged upon RU486 administration (the inducer of *elav-GS-GAL4*), whereas it is significantly reduced under starvation conditions. Together with the control data (*elav-GS-GAL4>UAS-Luc-RNAi*) presented in Figs. 2A-C, these results further indicate that CREB is critical for starvation-induced sleep changes.

In response to the distribution of data in Figure 3E, I still would have expected these data to be continuous, and the authors' explanation that it is the result of rounding is not convincing without details of their rounding methodology. The authors should explain their rounding to ensure reproducibility, and/or include the raw data so that one can "assess the rigor of the analysis".
In Figure 3E, cAMP levels were measured using a FRET-based reporter. We have now provided the detailed procedures in the Methods section on **lines 286-290**. The raw data also have been included in supplementary file.
Shapiro-Wilk normality tests performed in GraphPad Prism 10 yielded p-values greater than 0.05, confirming that the datasets did not deviate significantly from normality. Following reanalysis, we consider the segmented distribution more likely attributable to sample size than to rounding, as rounding did not substantially alter the distribution.

The authors seem to have made a copy-paste mistake. I believe they pasted a response to reviewers in the methods section "GeneSwitch-mediated transgene expression" (that section includes the phrase "We have updated the Methods section and the figure legends accordingly").
Thank you for pointing this out. We have deleted it accordingly. We once again greatly appreciate the reviewer's valuable comments, which have helped improve our manuscript.

Third decision letter

MS ID#: bio.062570R2

MS Title: CREB promotes starvation-induced sleep loss by regulating octopamine production in Drosophila

Authors: Shiye Zhu, Zhian Rao, Youjie Yin, Shuxin Chen and Hansong Deng

I am happy to tell you that your manuscript has been accepted for publication in Biology Open, pending our standard publication integrity checks. Reviewers have a few minor comments, please address those before the final version goes online. It was accepted on 14th July 2026.

© 2026. Published by The Company of Biologists under the terms of the Creative Commons Attribution License (https://creativecommons.org/licenses/by/4.0/).
